# Transfusion with Preserved Red Blood Cells

**Published:** 1918-08

**Authors:** 


***Transfusion with Preserved Red Blood Cells.*** By OSWALD H. ROBERTSON, Captain. M. R. C., *British Medical Journal*, June 22, 1918.

The author in his summary says : as first found by Rous and Turner it is possible to preserve living human red blood cells for several weeks in a solution of dextrose and citrate when kept at ice-box temperature. This method of keeping blood has been made use of recently for giving transfusions at casualty clearing stations during a rush period. A quantity of blood was stored up beforehand ready for use when needed. The blood was kept for varying periods up to twenty-six days before transfusion. Twenty-two transfusions were given in twenty cases of hemorrhage. The results of preserved blood transfusion were quite as striking as those seen after transfusion with blood freshly drawn. There was the same marked improvement, the patients stood the operation well and subsequent progress was quite as good as in those cases transfused by the usual methods. The introduction of kept red blood cells had no apparent harmful effect as there were no reactions or evidence of increased hemolysis after transfusion. The transfusion can be given relatively quickly and the technique, which is simple and easily acquired, can be carried out by one medical officer. Experiments in the transportation of preserved blood have shown that it can be carried a considerable distance without injury.

